# Left side gallbladder with agenesis of right anterior sector and absence of right hepatic duct. A case report

**DOI:** 10.1016/j.ijscr.2019.06.034

**Published:** 2019-06-21

**Authors:** Filippo Banchini, Ekerette Francis Ekpo, Luigi Conti, Patrizio Capelli

**Affiliations:** a Department of General Surgery, Guglielmo da Saliceto Hospital, Piacenza, Italy; b Plateau State Hospitals Pankshin in Plateau State, Nigeria

**Keywords:** Left gallbladder, Gallbladder, Anomaly, Biliary, Agenesis, Liver

## Abstract

**Introduction:**

Left sided gallbladder is a rare congenital abnormality more often discovered as incidental finding during laparoscopic cholecystectomy requiring surgical challenge to prevent complication. We present a case of a true left sided gallbladder treated with laparoscopic cholecystectomy.

**Case:**

A 90 y/o woman with acute abdominal pain was hospitalised and underwent a laparoscopic cholecystectomy. A preoperative CT scan didn't show the variation. Intraoperative finding revealed a true left sided gallbladder that was carefully dissected at the Calot's triangle with identification of the cystic duct and artery. After a complete separation of the gallbladder from the liver, the cystic duct and the artery were safely clipped and transected. CT scan reconstruction reveal agenesis of the right superior sector with simultaneous absence of the right biliary duct.

**Discussion:**

True left sided gallbladder is defined as one attached in the left of the ligamentum teres and falciform ligament. This abnormality predispose to a numerous anatomical variation. Unfortunately its finding is more often incidentally during intervention and recognition of such variation could be difficult that can be of high risk for surgical injury.

**Conclusion:**

Left sided gallbladder presents a significant challenge to the surgeon and makes it even more difficult to avoid postoperative complications. Therefore, a careful dissection of Calot’s triangle and the complete mobilisation of the gallbladder should be done before clipping the cystic duct and artery. However, if a preoperative finding suggests a left sided gallbladder, then an in-depth preoperative anatomical study is advocated.

## Highlights

• Left side gallbladder is more often discovered intraoperatively.
• A lot of anatomical variations are described.
• The three-dimensional reconstruction can better highlight intra and extraparenchimal anatomy.
• The knowledge of this anomaly could be useful both in cholecystectomy technique and liver resection.

## 1 Introduction

Left sided gallbladder is a rare anatomical variation with a prevalence of 0.1-0.7%. It comprises the fusion of the gallbladder itself on the left side of the liver with consequent anatomical modification both biliary and portal systems. More often it is an intraoperative finding that requires the surgeon’s meticulous attention in dissection in order to prevent complications. We present a rare case of left sided gallbladder with agenesis of the right superior sector of the liver with simultaneous intrahepatic biliary modification which was treated in our institution with the laparoscopic approach. This work has been reported in line with the SCARE criteria [1].

## 2 Case Report

**Fig. 1:**
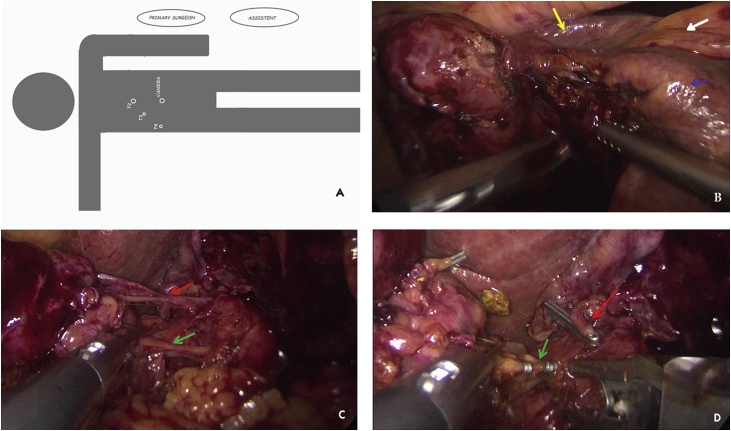
A) Surgeon and Trocar position; B) Image of left side gallbladder with, white arrow: round ligament; blue arrow: left lobe; green arrow: gallbladder attached to left lobe; yellow arrow: right lobe at the umbilical fossa; C) Complete dissection of the gallbladder with visualisation of cystic duct (green arrow) and cystic artery (red arrow); D) Safe clipping and section of the cystic artery (red arrow) and cystic duct (green arrow) visualised on the right side of the common bile duct.

**Fig. 2:**
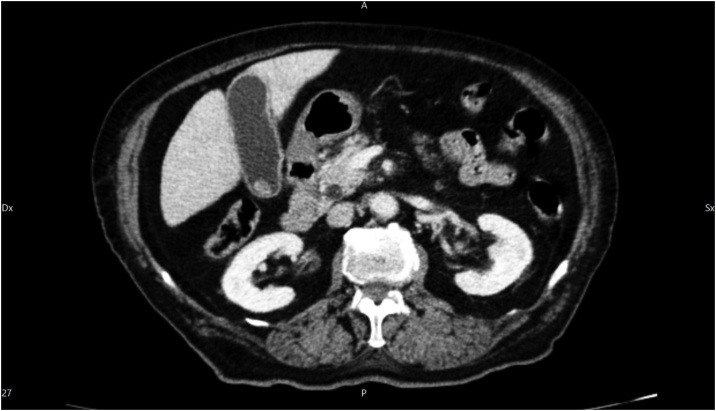
CT scan : gallbladder between left lobe and right lobe.

**Fig. 3:**
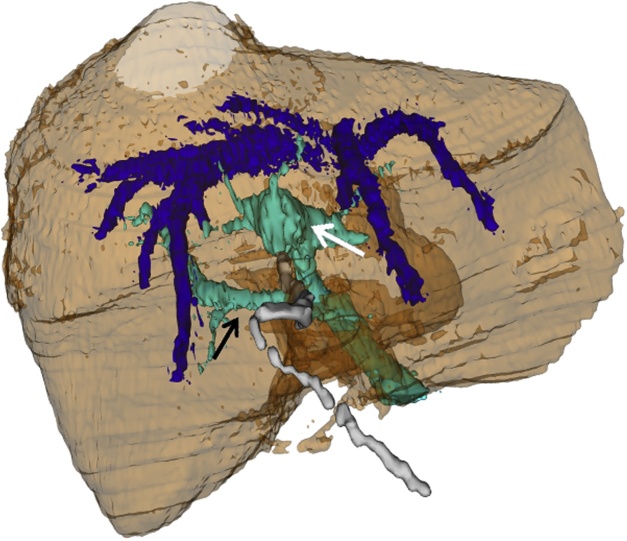
Three-dimensional reconstruction of the portal system: white arrow left portal branch; Black arrow: right posterior portal branch; absence of the branch for the right anterior sector between the two arrowed branches.

**Fig. 4:**
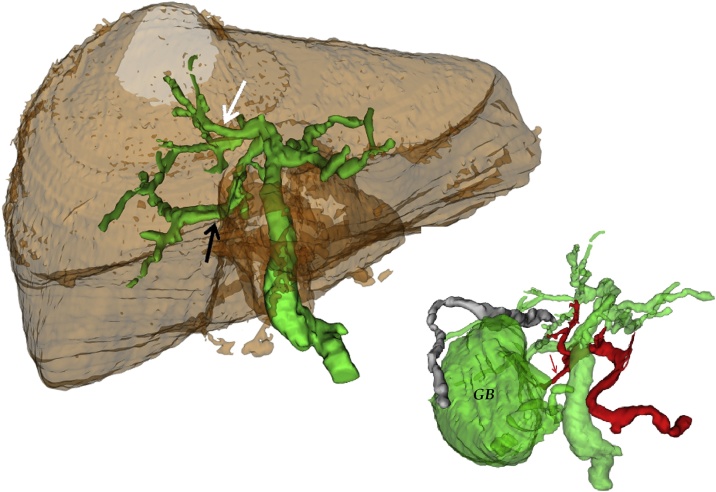
Three-dimensional reconstruction of the biliary system: white arrow branch for segment four; black arrow: branch for right posterior sector; GB: gallbladder with cystic duct on the right side of the common bile duct; Red arrow: cystic artery passing behind the common bile duct.

A diabetic 90 years old woman with cough and diffuse abdominal pain was admitted into our hospital with an initial diagnosis of sepsis. Blood sample investigation revealed mild leucocytosis with PRC augmentation and normal AST, ALT and bilirubin levels. Preoperative ultrasound revealed thickened gallbladder wall suggestive of acute cholecystitis. She has had previous sigmoid resection for diverticulitis and ERCP for choledocholithiasis without any mention of biliary abnormality. She was scheduled for a laparoscopic cholecystectomy during which the incidental intraoperative finding of a left sided gallbladder was made. Standard trocar positions were used (Fig. 1A) without any variation of technique, simply following the Strasberg criteria for safe cholecystectomy [2]. Having seen the anatomical variation (Fig. 1B), a careful dissection of Calot’s triangle was carried out, the cystic duct and artery were identified and isolated, and subsequent complete dissection of the gallbladder from the liver was done (Fig. 1C). This manoeuvre allowed us to demonstrate a right sided positioning of the cystic duct. Furthermore, a safe clipping and transection of the cystic duct and artery was achieved (Fig. 1D), and the gallbladder was extracted in an endobag. The postoperative course was complicated by an aggravation of diabetes and basal pneumonia which were controlled postoperatively in the intensive unit. The patient was discharged on postoperative day 16, in good condition. Retrospective analysis of the clinical case was done and a previous CT scan was reevaluated. Even with the knowledge of a left sided gallbladder, the CT scan was unable to demonstrate its attachment to the left side of the liver. This was probably due to the presence of a hypertrophic left lobe mimicking only a contiguity with the gallbladder, which itself appeared to be in a physiological position (Fig. 2). A vascular and biliary reconstruction was then evaluated with evidence of agenesis of the right anterior sector of the liver (Fig. 3) with the absence of the right hepatic duct and an intrahepatic variation with insertion of the right inferior sectorial duct into the left common duct (Fig. 4) as type A3 of Huang Classification [3].

## 3 Discussion

Left sided gallbladder is infrequent and is characterized by the fusion of the gallbladder to the left of the round ligament. As suggested by some authors [4] it is more often discovered intraoperatively and this situation can lead to complications for the patient and some difficulty with resolving them. In fact, as the first reported case by Hochstetter in 1886 [5] a lot of anatomical variations were described involving both the portal system and the biliary tree, and its presence in situs viscerus inversus. The gallbladder can also presents a number of congenital variations as agenesis, multiseptate and duplicated gallbladder or can be displaced intrahepatic. Portal abnormality was described by Kawai [6] with agenesis of segment 4. In our case, a kind of fusion between the right and the left anterior sectors was seen with simultaneous portal and biliary variations and agenesis of the right anterior portal sector. The 4th segment received the arterial supply from the right hepatic artery and the portal and biliary structures from the Umbilical Fissure and the gallbladder presented normal origine of cystic duct and cystic artery at right side of the hepatic pedicle (Fig. 4). This particular variation could be useful in case of a liver resection. More interesting for the clinical situation of cholecystectomy, and even in liver resection, are the abnormalities of the biliary tree. To understand that, it is important to make a distinction between false left sided gallbladder and the true one as described by various authors. A detailed definition of true left sided gallbladder is the one that is found at the base of segment III and to the left of the ligamentum teres and falciform ligament and the cystic artery which crosses in front of the CBD from right to left. The cystic duct may join at either side of the common hepatic duct or even join the left hepatic duct directly [7]. In false left sided gallbladder, hilar and pedicle structures are always in their normal position with the gallbladder only being attached to the left side of the liver, whereas in true left sided gallbladder, embryological evolution could move hilar structures towards the left side of the hepatic pedicle which may result in the rotation and modification of the cystic insertion on the left bile duct as well as variations of the common bile duct in the form of a duplication [8] or infraportal bile duct positioning [9]. It is evident that the presence of such an abnormality could give rise to disparities in the intraparenchymal structures. In our case simultaneous portal and biliary modification were seen with agenesis of the right anterior sector, absence of the right hepatic duct, a kind of fusion of the portal branches in the umbilical portion of left portal vein and insertion of the right inferior sector duct in the left hepatic duct. Considering the anomaly of left sided gallbladder, in the case of a fortuitous left side cholecystectomy, a careful dissection must be done while sticking to the Strasberg criteria [1] and, as done in our case, dissecting the whole gallbladder from the liver before clipping of the cystic structures. Alternatively, intraoperative cholangiography could be useful in suspicious biliary abnormality of cystic insertion. However, if left sided gallbladder is suspected preoperatively, especially when liver surgery is necessary, an anatomical study with cholangiography and CT scan should be considered.

## 4 Conclusion

Left sided gallbladder is a rare anatomical variation, more often an incidental discovery during laparoscopic cholecystectomy. This modification predisposes to a number of portal and biliary abnormalities most of which are difficult to recognize intraoperatively. Careful dissection must be done in Calot's triangle and, in order to prevent injury to the common hepatic duct, we advocate that a dissection of the entire gallbladder from the liver should be done before clipping the cystic duct and the artery. The importance of intrahepatic and hilar variations remain crucial in liver surgery, and a preoperative knowledge of a left sided gallbladder calls for in-depth study of the anatomy itself by cholangiography and CT scan.

## Conflicts of interest

All the authors declare no conflict of interest.

## Sources of funding

All authors declare no source of funding.

## Ethical approval

The clinical case is exempt from ethical approval.

## Consent

Informed consent signed by a daughter of the patient to publish the article. The patient is unable to sign at this moment.

## Author’s contribution

Dott Banchini Filippo: wrote the paper, study concept or design, data collection, data analysis and interpretation, image reconstruction.

Dott Ekpo Ekerette Francis: contribute to interpretation, revision, data collection and data analysis.

Dott Conti Luigi: contribute to data collection and interpretation.

Dott Banchini Filippo: performed the surgery.

Dott Capelli Patrizio: approved the paper.

All authors have approved the final article for submission.

## Registration of research studies

No registry is required for clinical case.

## Guarantor

Guarantor is Dott Banchini Filippo.

## Provenance and peer review

Not commissioned, externally peer-reviewed.

## References

[1] Agha R.A., Borrelli M.R., Farwana R., Koshy K., Fowler A., Orgill D.P., For the SCARE Group (2018). The SCARE 2018 statement: updating consensus Surgical Case REport (SCARE) guidelines. Int. J. Surg..

[2] Strasberg Steven M., Michael Brunt L. (2010). Rationale and use of the critical view of safety in laparoscopic cholecystectomy. J. Am. Coll. Surg..

[3] Huang T.L., Cheng Y.F., Chen C.L., Lee T.Y. (1996). Variants of the bile ducts: clinical application in the potential donor of living related hepatic transplantation. Transplant. Proc..

[4] Nagendram S., Lynes K., Hamade A. (2017). A case report on a left sided gallbladder: a rare finding during cholecystectomy. Int. J. Surg. Case Rep..

[5] Hochstetter F. (1886). Anomaliem der pfortader und der nabelvene in verbindung mit defect oder Linkscage der gall-en-blase. Arch. Anat. Entwick..

[6] Kawai R., Miyata K., Yuasa N., Takeuchi E., Goto Y., Miyake H., Nagai H., Hattori M., Imura J., Hayashi Y., Kawakami J., Kobayashi Y. (2012). True left-sided gallbladder with a portal anomaly: report of a case. Surg. Today.

[7] Alharthi S., Bernon M., Krige J.E.J. (2012). Beware the left-sided gallbladder. S. Afr. J. Surg..

[8] Bender Elizabeth A., Springhetti Sara, Shemisa Kamal, Wittenauer Justine (2007). Left-sided gallbladder (Sinistroposition) with duplication of the common bile duct. JSLS.

[9] Ishii Hiromichi, Noguchi Akinori, Onishi Mie, Takao Koji, Maruyama Takahiro, Taiyoh Hiroaki, Araki Yasunobu, Shimizu Takeshi, Izumi Hiroyuki, Tani Naoki, Yamaguchi Masahide, Yamane Tetsuro (2015). True left-sided gallbladder with variations of bile duct and cholecystic vein. World J. Gastroenterol..

